# Genetic linkage study of high-grade myopia in a Hutterite population from South Dakota

**Published:** 2007-02-15

**Authors:** Sudha Nallasamy, Prasuna C. Paluru, Marcella Devoto, Nora F. Wasserman, Jie Zhou, Terri L. Young

**Affiliations:** 1 Divisions of Ophthalmology, Children’s Hospital of Philadelphia, Philadelphia, PA; 2 Human Genetics, Children's Hospital of Philadelphia, Philadelphia, PA; 3 University of Pennsylvania School of Medicine, Philadelphia, PA; 4 Center for Clinical Epidemiology and Biostatistics (CCEB), University of Pennsylvania School of Medicine, Philadelphia, PA; 5 Dipartimento di Medicina Sperimentale e Patologia, Universita' La Sapienza, Roma, Italy; 6 Departments of Ophthalmology and Pediatrics, Duke University Medical Center, Durham, NC

## Abstract

**Purpose:**

Myopia is a common, complex disorder, and severe forms have implications for blindness due to increased risk of premature cataracts, glaucoma, retinal detachment, and macular degeneration. Autosomal dominant (AD) non-syndromic high-grade myopia has been mapped to chromosomes 18p11.31, 12q21-23, 17q21-23, 7q36, 2q37.1, 7p15.3, 15q12-13, 3q26, 4q12, 8p23, 4q22-q27, 1p36, and Xq23-q25. Here, we demonstrate evidence of linkage for AD non-syndromic high-grade myopia in a large Hutterite family to a locus on chromosome 10q21.1.

**Methods:**

After clinical evaluation, genomic DNA was genotyped from 29 members of a Hutterite family from South Dakota (7 affected). The average refractive error of affected individuals was -7.04 diopters. Microsatellite markers were used to exclude linkage to the known AD nonsyndromic high-grade myopia loci as well as to syndromic high-grade myopia loci. A genome screen was then performed using 382 markers with an average inter-marker distance of 10 cM followed by fine-point mapping in all regions of the genome that gave positive LOD scores. SimWalk2 software was used for multipoint linkage based on AD and autosomal recessive (AR) models with a penetrance of 90% and a disease allele frequency of 0.001.

**Results:**

A maximum multipoint LOD score of 3.22 was achieved under an AD model at microsatellite marker *D10S1643*. Fine point mapping and haplotype analysis defined a critical region of 2.67 cM on chromosome 10q21.1. Haplotype analysis demonstrated two distinct haplotypes segregating with high-grade myopia, indicative of two distinct mutations occurring in the same gene.

**Conclusions:**

We have identified a presumptive myopia locus for high-grade myopia based on linkage and haplotype analysis.

## Introduction

Myopia, or nearsightedness, affects approximately 25% of the adult population in the United States [1-5], and is increasing in prevalence. High-grade myopia (<5 diopters) affects 4.5% of the U.S. population 40 years and older [5], and is a major cause of legal blindness in many developed countries [6-8], as it predisposes individuals to premature cataracts, glaucoma, retinal detachment, and macular degeneration [9-14]. Though myopia affects a significant proportion of the population in many countries, it is reaching epidemic proportions in some Asian countries. Taiwan, for example, has a myopia prevalence of 84% among 16- to 18-year-old high school students and a high-grade myopia prevalence of 21% among 18-year-old students [15].

Determining the role of genetic factors in the development of non-syndromic myopia has been influenced by the high prevalence, genetic heterogeneity, and clinical spectrum of this condition, as well as possible modulating environmental effects. However, in the past few years, considerable progress has been made. An X-linked recessive form of myopia was designated the first myopia locus (MYP1, OMIM 310460) on chromosome Xq28 [16]. Reanalysis of this Danish pedigree and another X-linked pedigree of Danish descent suggests that this disease locus involves a cone dysfunction and not simple myopia [17]. By studying several medium to large multigenerational families with autosomal dominant (AD) high-grade myopia, we have found significant linkage at chromosome 18p11.31 (MYP2, OMIM 160700) [18], chromosome 12q23.1-24 (MYP3, OMIM 603221) [19], chromosome 17q21-23 (MYP5, OMIM 608474) [20], and most recently at chromosome 2q37.1 (MYP12, OMIM 609994) [21]. In addition, other research groups studying AD high-grade myopia have found suggestive linkage on chromosome 7q36 (MYP4, OMIM 608367) [22] and significant linkage to chromosomes 7p15.3 [23], chromosome 15q12-13 [24], and chromosome 4q22-q27 (MYP11, OMIM 609994) [25]. Two recent studies have determined loci for common myopia: one in an Ashkenazi Jewish cohort with linkage to chromosome 22q12 (MYP6, OMIM 608908) [26], chromosome 1p36 (MYP14, OMIM 610320) [27], and another in English dizygotic twins with linkage to the PAX6 gene region on chromosome 11p13 (MYP7, OMIM 609256), chromosome 3q26 (MYP8, OMIM 609257), 4q12 (MYP9, OMIM609257), and 8p23 (MYP10, OMIM 609259) [28]. Most recently X-linked recessive myopia was mapped to Xq23-25 (MYP13, OMIM 300613) region of the X-chromosome [29].

Herein, we study high myopia in a group of Hutterites from South Dakota. The Hutterites were originally refugees from the Anabaptists from Switzerland, Germany, and the Tyrol who settled in Moravia and were united under Jacob Hutter's leadership in the 1500s. The four most important Hutterian tenets were adult baptism of believers, community of goods, nonresistance, and the separation of church and state. Due to religious persecution, the Hutterites moved frequently through the centuries, and today about 25% of Hutterites live in the United States. The Hutterites have an internal educational system based within their colonies, starting instruction at age 6 years, and ending at 8th grade (age 13 years). In this study, we exclude linkage to known myopia loci, and report significant linkage of AD nonsyndromic high-grade myopia to a presumptive locus on chromosome 10q21.1 for a large, multi-generational Hutterite family. To our knowledge, this is the first time that high myopia has been studied in a genetically isolated group.

## Methods

### Subject data collection

A large Hutterite family from South Dakota (Pedigree MYO-101) with 29 members (7 affected) consented to participate in the study. This study was approved by the Institutional Review Board of The University of Minnesota and The Children's Hospital of Philadelphia, and adhered to the tenets of the Declaration of Helsinki. This family was selected based on the presence of affected individuals in multiple generations. Individuals with high-grade myopia, defined as a spherical refractive error of <-5.00 diopters (D) in at least one eye, were considered "affected" and individuals with a spherical refractive between -2.00 and -5.00 D in at least one eye were considered "unknown", as they had an increased likelihood of developing high-grade myopia. All other individuals (spherical refractive errors >-2.00 D in both eyes) were considered "not affected". No participant had known ocular disease or insult that could predispose to myopia or any known genetic syndrome associated with myopia. Glaucoma, keratoconus, corneal thinning, lenticonus, and dislocated lens were absent in study participants. The family did not display any other systemic genetic traits.

A comprehensive ophthalmic examination and blood collection was performed by one of the authors, TLY, as previously described [18]. Details of the ophthalmic examination are summarized in Table 1. DNA was isolated from peripheral blood lymphocytes using standard techniques (Gentra Systems, Minneapolis, MN).

**Table 1:** Clinical characteristics of pedigree MYO-101.

**Subject**	**Gender**	**Myopia phenotype**	**Age at onset**	**Age at exam**	**Refractive Error OD**	**Refractive Error OS**	**Ocular history**
7242	F	NA	40	56	+0.25 +0.75x79	+0.25 +0.50x77	---
7243	M	UK	14	32	-2.25 +0.25x90	-1.75 sphere	---
7244	M	NA	---	19	-0.25 sphere	Plano	---
7245	F	A	15	22	-3.75 +0.75x90	-5.25 +1.25x90	---
7246	F	UK	14	17	-3.00 sphere	-2.25 +0.25x110	---
7247	F	UK	14	15	-2.00 sphere	-1.50 +1.00x80	---
7248	M	UK	10	21	-2.00 +0.50x176	-2.75 +1.50x165	---
7249	M	NA	30	54	+2.50 sphere	+2.50 sphere	---
7251	F	A	6	9	-5.50 +0.75x95	-6.00 +1.25x90	---
7252	F	A	7	12	-8.00 +1.25x90	-6.50 +0.25x90	---
7253	F	A	14	60	-11.25 +0.75x105	-12.25 +0.75x45	---
7254	M	A	16	31	-5.50 +0.50x155	-9.00 +3.50x60	---
7255	M	NA	45	48	-0.50 +0.25x61	-0.25 +0.50x90	---
7256	M	NA	---	32	-0.50 sphere	+0.25 sphere	---
7257	M	NA	---	26	-0.75 +1.00x75	-0.25 +0.25x95	---
7258	F	UK	14	33	-3.00 +1.25x90	-1.25 +0.75x90	---
7259	M	NA	17	33	-1.25 -1.25x125	-1.50 -0.75x50	---
7260	M	NA	30	69	+0.50 +1.00x9	-1.25 +0.50x16	---
7261	F	NA	15	59	+1.25 +0.25x2	+1.00 +0.50x165	---
7262	M	NA	15	59	-0.75 +1.00x8	-0.25 +0.50x174	---
7263	M	UK	10	24	-1.00 +0.50x75	-2.50 +1.25x80	---
7264	F	UK	19	47	-2.75 +1.25x37	-2.00 +0.75x150	---
7266	F	UK	15	55	-3.00 +1.25x30	-2.5 +0.75x145	---
7268	F	UK	9	54	-4.25 +2.5x85	-3.25 +2.75x70	---
7270	M	NA	---	9	Plano	Plano	---
7272	M	A	13	31	-4.25 +1.00x180	-4.25 +1.00x164	s/p LASIK
7274	M	A	14	37	-6.75 +3.75x90	-4.75 +1.75x75	---
7275	M	NA	50	61	+0.5 +0.25x140	Plano+0.75x170	---
7277	M	NA	---	35	+0.25 +0.50x79	+0.25 +0.25x105	---

Clinical characteristics of pedigree MYO-101. A=Affected, NA= Not Affected, UK= Unknown, M= Male, F=Female, OD= right eye, OS= left eye, Plano=no refractive error.

### Candidate loci screening

Initially, we screened 15 candidate gene loci using intragenic and flanking microsatellite markers. These loci included all of the known myopia loci mentioned earlier, except for Xq28, as there was no evidence for an X-linked mode of inheritance. The other candidate regions screened were syndromic loci with high-grade myopia as a consistent phenotype, including Stickler syndrome types 1, 2, and 3; Marfan syndrome; juvenile glaucoma; and Knobloch syndrome. Of these syndromes, all are inherited in an autosomal dominant fashion except for Knobloch syndrome, which is autosomal recessive (AR). As there were many unknown refractive errors (family members not examined or genotyped) in the older generations, we were initially uncertain whether the mode of inheritance was AD or AR.

### Genotyping

Genome screening was performed on Pedigree MYO-101 using 382 microsatellite markers from a commercial set (ABI Prism Linkage Mapping Set MD-10; Applied Biosystems, Inc., Foster City, CA) with an average inter-marker distance of 10 cM. The PCR reactions were prepared in 10 μl volumes in 96-well plates with 6 μl of True Allele PCR Premix, 1 μl primer, 1 μl sterile H_2_O, and 2 μl of 3 μg/μl DNA. The conditions recommended by Applied Biosystems were optimized for our PCR machines. The fluorescently labeled PCR products were diluted and pooled (3-7 markers/pool) and run on an automated DNA sequencer (ABI Prism 3730 DNA Analyzer; Applied Biosystems). GeneMapper Software Version 3.0 (Applied Biosystems) was used for automated allele calling, visualization of alleles, and manual genotype verification. After the initial genome screen, markers from the ABI Prism Linkage Mapping Set HD-5 (with an average inter-marker distance of 5 cM) and additional custom-made markers selected from publicly available genetic maps [30,31] were added in all regions with positive LOD scores. Inter-marker recombination frequencies and marker order were obtained from the standard marker databases of the Marshfield Center for Medical Genetics, Gnthon, and the UCSC (University of California Santa Cruz) database.

### Linkage analysis

Mendelian error checking was performed using PEDMANAGER Version 0.9 software. Mega2 Version 3.0 software [32] was used to create the necessary files required for statistical analysis. SimWalk2 Version 2.89 software [33-35] was used for multipoint linkage analysis based on both AD and AR models, as the mode of inheritance was uncertain. A 90% penetrance and a myopia allele frequency of 0.001 were used. Marker allele frequencies were estimated by maximum likelihood based on our pedigree data. Two-point linkage analysis could not be performed because the available linkage analysis programs were not capable of handling the large size and complexity (many loops) of this pedigree.

### Positional candidate gene mutation screening

Protocadherin 15 (*PCDH15*) contains 33 exons and encodes a protein of 1,955 amino acids with a molecular mass of about 216 kDa [36]. *ZW10* interactor (*ZWINT*) contains 9 exons and encodes a protein of 277 amino acids with a molecular mass of 32 kDa [37]. We designed and optimized 36 sets of forward and reverse primer pairs for *PCDH15* and 8 for *ZWINT*. The primer pairs were constructed to extend 50 to 200 bp beyond each intron-exon boundary (Table 2). For each amplimer, PCR was performed on 150 ng of participant genomic DNA using *Taq* polymerase (Ampli*Taq* Gold; Roche Molecular Systems, Inc., Branchburg, NJ) at 55 °C annealing temperature. Amplified products were separated by agarose gel electrophoresis and visualized by staining with ethidium bromide. Two highly myopic family members (7254 and 7272) were screened, along with 2 unaffected (average spherical refractive error=-0.31 D) family members (7256 and 7257) and 1 external control (C009), a United States caucasian male with no refractive error. If a polymorphism appeared to follow affection status, four additional affected individuals (7245, 7251, 7252, and 7274) were screened for that polymorphism in an attempt to confirm or exclude it as a myopia-causative mutation. Amplicons were sequenced using the BigDye® Terminator v3.1 Cycle Sequencing Kit and were run on an ABI 3730 DNA Analyzer (Applied Biosystems, Foster City, CA). Chromatograms were trimmed for quality and aligned with reference gene sequences from NCBI using Sequencher^TM^ (Gene Codes, Ann Arbor, MI).

**Table 2:** Primers designed for mutation screening.

**Exon**	**Forward Primer**	**Reverse Primer**
PCDH15		
1	TCACTTGCAATCATTACTACTCTAT	GGCTGACGAACACAGTACAATA
2	AAAAATCCTGGCTCGCTCTA	AATGTGGCACAGGTAATTAACTATG
3	TTATTCCAAGTAAAGCATGA	TTTTCCTTTTTAAATATTAATATAC
4	AAATGAGTTAAAGAAACTGTTGT	TATTTTCTGTCTTATGCTAGATATA
5	GTTTTCTTGATATTTCTAAACAGTT	TGTACTGAATGAACGAGTGCT
6	AATAATAGTATCATTACTAATCTGG	GAGCTGATTTTTATGCTGTAACA
7	ATGAAGAAGTGAGTGGCACA	TTCTATTTTCAGATGATAAGCATGT
8	AAAATAAAATAACCATGTTGGACT	TTTTTGTTGTTGTTTTTTGAGACTG
9	AAATATTTCCTTGGAATTGA	TGAGTTTACTGTCTCAGACGTTATA
10	TAACATAAAACTGCCTACAGTAGCG	TTTGTTCCTCAGCTGATGAAGGG
11	TTAATTCTCTCTTCTCTAGCTTATG	ATTAGTTAAAAAGAGAACCCTATA
12	CCAGACATCTCTTTCAGTTCCATAC	TCTTAATACTTTCACGTGGAT
13	TAATTTCTTCATGAGCATATCGTAT	CCTTTCAAATTGTAAGATTACCTG
14	AAGAATACTTGCCGCGCTTC	TTGTTGGGGGAAAAAACGACATGGT
15	CAGATGGAATTTTCATTCACTAGAG	AACAACTGAATAAACAATGCAGTC
16	AGAAAACAGAAAGGGAAGTACAAC	GATACGAAATGTTTGATTTTACACC
17	TGATTACTGAGAGGGGAAA	TGTCAAGTCAAAGGTTGCAGC
18	TTCAGATAGACAAATGCCAGAA	AGTCTTGAAGAATATCCAGCACA
19	ATTGTCATTGTCATTTCCTCCTT	GGCACACAAACCCTAATAGCA
20	GTCCTCGTTAAATTGATGCTGT	TTGGAAAATCTATGTTATGGGTC
21	AATTGAGAGGATGGAAGGCT	TTGCTGTCTTGTGATTCGGTC
22	CCCAAAGCAACCATTAATTTTTC	ATGAGGATAGAGATCAAAACGCA
23	CACCTGGTAGGCAGTGAGGAAAT	TTGAAACCCCAAAATATAATGT
24	ATGAATGTGTGAGGGCAGAGGGT	CAAATGAGGAAAAGGGCAAGGT
25	TTAGCGAAGTATTGATGTTGA	TCCCAAGGTTTATCCAGAG
26	AAGCATCATTATCAGTGTCCC	CCTACTGGAGGAATACAAACC
27	CCCCTCCGTCTAGGCTACTGATA	AAACTGAAAACACTGACCTATGGCT
28	GTAGGGAAGAGGTAATGTGGG	AGACACTGAGGGCTCCAA
29	GGCTGTCATCATTTTTCATTT	TTCAGTAACTATTTGGTGGGT
30	GCTTGTGTTTCAATTAGAGAA	CGCAAAGCCATGTTAC
31	GAATGACTCTAACGGTAACGAT	TGGAAGCTATAGGCAAACTATCT
32	CCTGTGGGGATGCTTTTGTAA	AATTTCTGGTTTGGTTTTGGC
33-1	AACTTGTTTCCTTACATTTCA	CCTTGCCTTATTTCCT
33-2	AAGAGGTAGCAGCAATCCATT	CGGCAGGCATCAAGTT
33-3	CCCTCCACCTCCTTCAG	TGGTTTAAGTTGGGTATCTAA
33-4	TTTTAGTGGGAATATGTGGG	AAAAAATTTAAATTAGGGAGATGAT
ZWINT		
1	GTGGATGTGGGGAGCGGCGAACGG	AAGAACTTCCACAGGCGCGCGGTCGAG
2 and 3	GTCTTCAGAAGCCAAGGGGTCG	CCCTGCTGATTTTCCTTTTGGCACGTA
3 and 4	GGCCATTTGTTTCTACAGATAAGCAAG	GTGGTGGTATGTACAAGATGAGAGCGA
5	TCAAAGGAGGAAGCAAAACGGTTTTC	CCAAGGTACACCCAGGGGTCTTGAGTA
6 and 7	CAAATTTACATCTCCAGCAGGTTGTAG	GCAAGAATGCCTCACCCTAGAAC
8	GAGCTCACTGTTATAATTCTGGTTCAC	AGGTAAATGAGAGATGGAACAGG
9a	CTTTAAATAACTTGAGACATTGCTAAC	TAATAAACTTTGAAATTTGGTGAGT
9b	AAATGCATTCTTTCCAAACCTATGTGA	GGTTTGAACACAGGGTGTGCAG

Forward and reverse primers designed for *PCDH15* and *ZWINT* for mutation screening.

## Results

**Figure 1:**
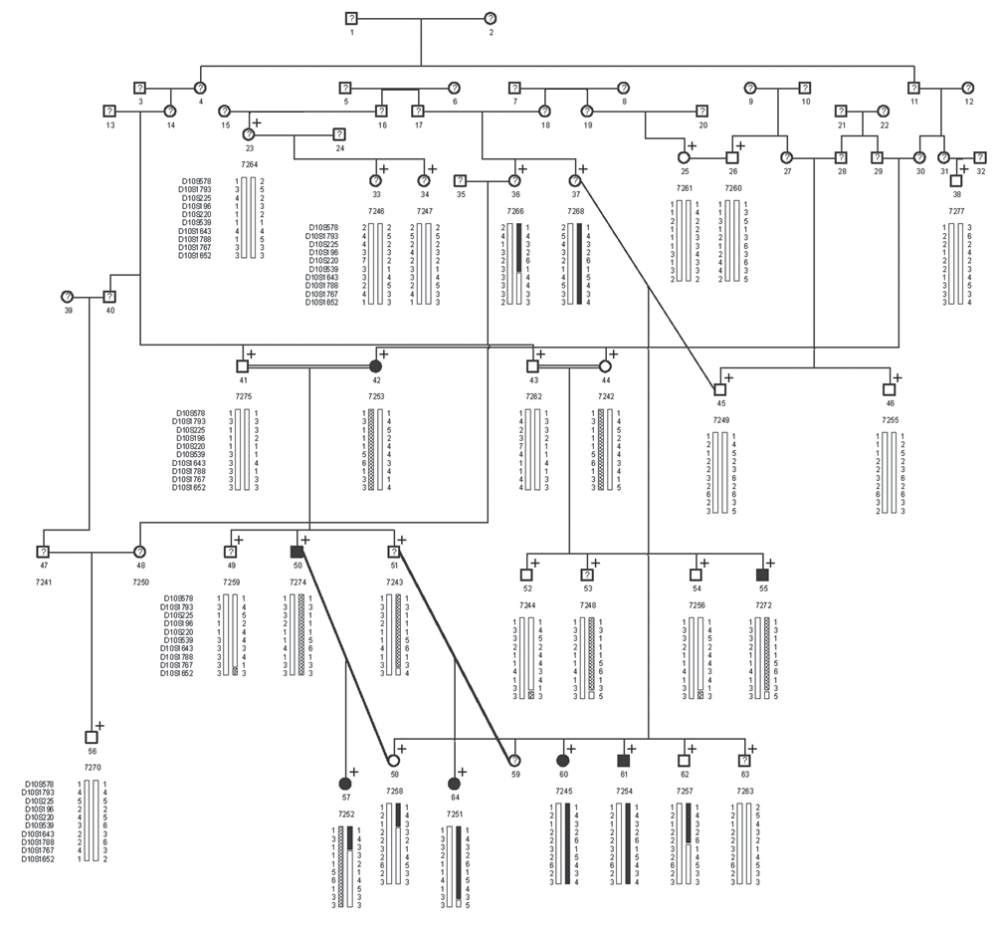
Pedigree MYO-101 with familial high-grade myopia. Circles and squares indicate females and males, respectively, while solid symbols show affected individuals. The alleles for the most informative polymorphic markers are shown for each studied individual. Haplotypes were constructed based on the minimum number of recombinations between these markers. The solid and crosshatched bars indicate the two affected haplotypes.

A multi-generational Hutterite family from South Dakota (MYO-101) with high-grade myopia was identified and characterized (Figure 1). The average spherical refractive error of affected individuals of Pedigree MYO-101 (excluding 7272, who had only post-LASIK refractive error information available) was -7.04 diopters (range, -3.75 to -12.25). Although we were not able to obtain pre-LASIK refractive error information for individual 7272, we made an assumption that he was an affected individual for purposes of analysis based on his self-report that he had twice the degree of myopia in diopters prior to his surgery. Two patients from family 101 (7245 and 7274) were anisometropic, with one highly myopic and one moderately myopic eye. The average spherical refractive error for "unknown" individuals was -2.39 D. All known syndromic and non-syndromic myopia loci were excluded in this family. The LOD scores at q=0.0 were as follows: *D18S63* (MYP2), -5.232; *D12S78* (MYP3), -0.221; *D7S2465* (MYP4), -5.781; *D17S944* (MYP5), -3.418; *D22S280* (MYP6), -0.960; *D11S904* (MYP7), -0.979; *D2S206* (2q37.1), -2.006; *D7S507* (7p15.3), -7.573; *D15S165* (15q12-13), -3.969; *D12S85* (Stickler syndrome type 1), -4.652; *D1S206* (Stickler syndrome type 2), -1.175; *D6S276* (Stickler syndrome type 3), -2.788; *D15S117* (Marfan syndrome), -5.464; *D1S218* (juvenile glaucoma), -3.131; *D21S1897* (Knobloch syndrome), -8.724.

**Figure 2:**
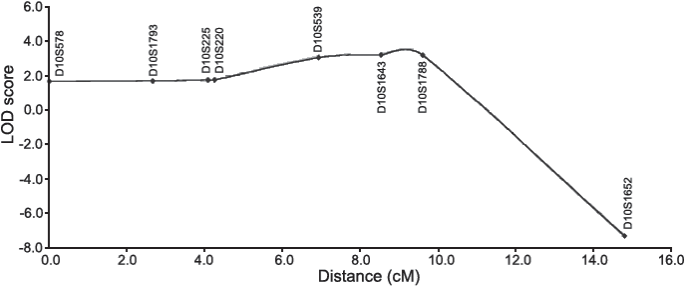
Multipoint LOD score data for pedigree MYO-101 for chromosome 10q21.1 with markers labeled along the X-axis. LOD scores were plotted against marker distance in centiMorgans (cM).

**Figure 3:**
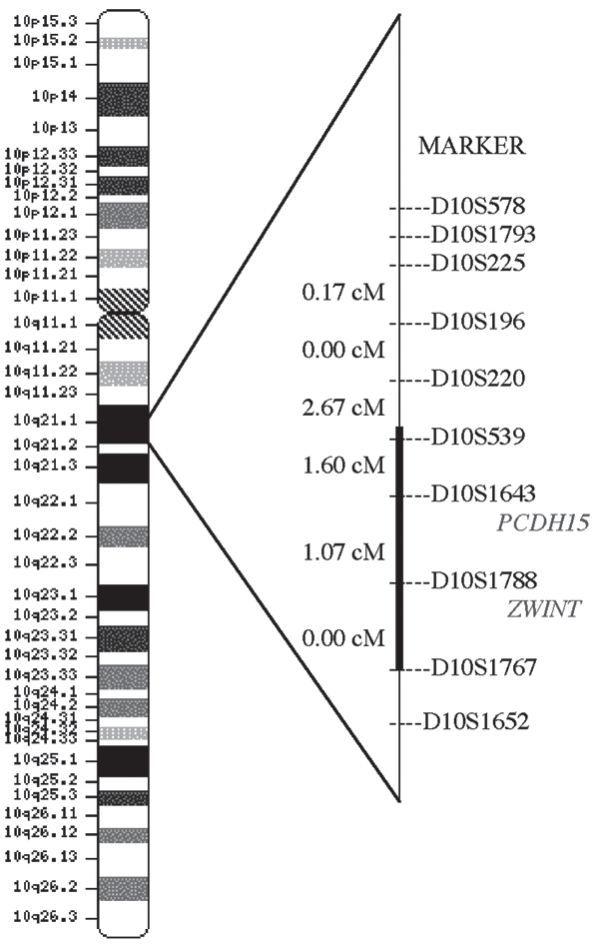
Schematic representation of a linkage map of microsatillite markers located at 10q21.1. The mapping order and genetic distances (in centiMorgans [cM]) were primarily obtained from the human genetic map of the Marshfield Center for Medical Genetics. The bold segment denotes the linked interval determined by multipoint linkage and haplotype analysis.

The multipoint analysis for the initial 10 cM genome screen resulted in no LOD scores >1.00 under an AR model. However, under an AD model, the multipoint linkage analysis resulted in four LOD score peaks above 1.00, occurring on chromosomes 1p36.32-1p36.31 (LOD=1.207), 1p36.11 (1.131), 9p24.1 (1.095), and 10p11.22-10q11.23 (2.108). Additional flanking microsatellite markers were then genotyped in all of these regions. The LOD scores for the chromosomes 1p and 9p regions reduced or increased negligibly. Haplotype analysis of the 1p36.11 and the 9p24.1 region did not reveal a consistent haplotype segregating with affected individuals. Haplotype analysis of the 1p36.32-1p36.31 region (max LOD score with additional markers =1.330) revealed a consistent affected haplotype in one branch of the family, but not in the other branch. However, on chromosome 10, the additional markers downstream of the initial 10p region produced positive LOD scores without an increase in the maximum multipoint LOD score. Additional markers were used in this region, and yielded a multipoint LOD score under an AD model of 3.22 at marker *D10S1643* on chromosome 10q21.1. The one-unit support interval ranged from marker *D10S539* through marker *D10S1767*, a 2.67 cM region (Figure 2 and Figure 3). Haplotype analysis correlated well with the multipoint linkage results, as unaffected individual 7257 had a recombination event either between markers *D10S220* and *D10S539* or between markers *D10S539* and *D10S1643* (Figure 1). It is impossible to determine where the recombination event occurs due to an allele that is common to the affected and unaffected haplotypes at marker *D10S539*. Thus, this unaffected individual has the affected haplotype upstream of *D10S539*, excluding this region from containing the disease gene.

Interestingly, haplotype analysis also revealed two distinct haplotypes segregating with high-grade myopia (Figure 1), designated in solid black and in crosshatching. This implies that either the mutation is old and has recombined into different genetic backgrounds (haplotypes), or two distinct mutations are responsible for the high-grade myopia seen in this family [38]. Upon inspection of the pedigree, it is evident that there are two distinct branches joined by marriage, making the possibility of two distinct mutations likely. In addition to the seven "affected" individuals, three "unknown" individuals [7243, 7248, and 7268 (average spherical refractive error of -2.72 D)], as well as one unaffected individual (7242), carried either one of the affected haplotypes. Thus, the mutation(s) appear to show incomplete penetrance.

Two known genes were identified within the 2.67 cM region: protocadherin 15 (*PCDH15*) and *ZW10* interacter (*ZWINT*). Direct sequencing of these two genes identified a total of 23 polymorphisms, none of which segregated with the affection status (Table 3). There were 21 polymorphisms identified in *PCDH15*; 17 intronic, 2 missense, and 2 silent, out of which 13 were novel. There were 2 identified intronic polymorphisms in *ZWINT*, one of which was novel. All 14 novel polymorphisms have been submitted to the National Center for Biotechnology Information (NCBI) dbSNP database.

**Table 3:** Observed sequence polymorphisms in the protocadherin 15 (*PCDH15*) and *ZW10* interacter (*ZWINT*) genes.

**Genes**	**Position**	**nt at GenBank (minus strand)**	**nt changes**	**dbSNP**	**AA changes**	**Sample number (red=affected)**
PCDH15						
Exon 2	83 bp in Exon 2	T	T/G	rs11004439	Ser->Ala	C009, 7256
Intron 2	50 bp, 5' of Exon 3	T	G	rs10825347	-	C009, 7252, 7253
	50 bp, 5' of Exon 3	T	G/T	rs10825347	-	7254, 7245, 7251, 7274, 7256, 7257
Intron 3	4 bp, 3'of Exon3	A	A/G	Novel	-	7254, 7251
Intron 4	31 bp, 5'of Exon5	T	T/C	rs11594958	-	7256
Intron 7	9 bp, 5'of Exon8	C	C/T	rs10740579	-	C009, 7257, 7272
	9 bp, 5'of Exon8	C	T	rs10740579	-	7254, 7256
Exon 8	4 bp in Exonic	C	C/T	Novel	Ala->Thr	C009
Intron 9	57 bp, 3'of Exon8	T	G	rs10763098	-	7254
	57 bp, 3'of Exon8	T	G/T	rs10763098	-	C009, 7256, 7257, 7272
Intron 9	71 bp, of 5' Exon 9	G	A	Novel	-	7254, 7245
	71 bp, of 5' Exon 9	G	G/A	Novel	-	C009, 7256, 7257, 7252, 7251
Intron 13	23 bp, of3' Exon13	G	G/A	rs7093302	-	C009, 7256, 7257, 7252, 7274, 7251
Intron 14	43 bp, of5' Exon 15	-	Insertion A	Novel	-	C009, 7254, 7256, 7257, 7272
Intron 17	141 bp, 3' of Exon 17	C	C/T	Novel	-	7254, 7257
Intron 20	37 bp, 3' of Exon 20	C	C/T	Novel	-	7256, 7272
Exon 21	35 bp in Exon 21	G	G/A	rs2135720	Ile->Ile	7256, 7272
Intron 21	109 bp, 5' of Exon 22	T	T/C	Novel	-	C009, 7254
Intron 22	48 bp, 5' of Exon 23	G	G/A	rs2593107	-	C009, 7254
Intron 25	72 bp, 5' of Exon 26	A	A/G	Novel	-	C009, 7254
Intron 25	68 bp, 5' of Exon 26	T	T/G	Novel	-	7254, 7257
Intron 29	20 bp, 5' of Exon 30	C	C/T	Novel	-	C009, 7254, 7256, 7257, 7272
Intron 31	188 bp, 3' Exon 31	C	C/T	Novel	-	C009, 7254, 7256, 7257, 7272
Intron 32	120 bp, 3' of Exon 33	T	T/C	Novel	-	C009, 7254, 7256, 7257, 7272
Exon 33	215 bp, of Exon 33	C	C/A	Novel	Leu->Leu	7256, 7272
ZWINT						
Intron5	30 bp, 5' of Exon 4	C	C/T	Novel	-	7254, 7257
Intron 3	74 bp, 3' of Exon 3	G	A/A	rs3861049	-	C009, 7254, 7256, 7257, 7272

Affected individual sample numbers are in red. C009, external control; rs, public reference single nucleotide polymorphism (SNP) number from the dbSNP database. Note: Samples C009, 7254, 7256, 7257, and 7272 were sequenced for all exons. Additional samples were sequenced for exons with polymorphisms that initially appeared to segregate with high-grade myopia.

## Discussion

This study identifies a novel autosomal dominant locus for high-grade myopia and provides additional evidence for the genetic heterogeneity of myopia. Linkage analysis placed a gene for myopia susceptibility on chromosome 10q21.1, within a 2.67 cM interval. Linkage was excluded to the candidate gene regions for the Stickler syndromes, Marfan syndrome, juvenile glaucoma, and Knobloch syndrome, ensuring that this family did not exhibit a mild phenotypic expression of these conditions. Similarly, linkage was excluded to known autosomal myopia loci. Interestingly, two different haplotypes segregate with high-grade myopia in this family, suggesting the possibility that either the mutation is old and has recombined into different genetic backgrounds (haplotypes), or two distinct mutations are responsible for the high-grade myopia seen in this family.

A search for genes physically mapped between markers *D10S539* and *D10S1767* revealed only two known genes: *PCDH15* (protocadherin 15) and *ZWINT* (*ZW10* interactor). *PCDH15* is a member of the cadherin superfamily of calcium dependent cell-cell adhesion molecules. A 15 cM region encompassing the *PCDH15* gene region was initially implicated in autosomal recessive Usher syndrome in a 1997 study of a Hutterite family [39]. This region was further refined in a study of two Pakistani families segregating Usher syndrome type 1F that demonstrated two homozygous mutations (splice site acceptor mutation and nonsense mutation) in the 33 exon *PCDH15* gene [36]. Additional truncating mutations have since been discovered in other individuals with Usher syndrome. Usher syndrome is characterized by progressive pigmentary retinopathy and sensorineural hearing loss, and individuals with Usher syndrome type 1 have congenital severe-to-profound hearing loss and vestibular dysfunction. Interestingly, individuals with non-syndromic recessive hearing loss (*DFNB23*) have been found to have missense mutations in *PCDH15* [40]. Immunohistochemistry has demonstrated that *PCDH15* is expressed in the photoreceptors, especially in the outer photoreceptor segments of human and monkey retinas [40]. Cones demonstrated strong immunoreactivity and rods demonstrated a diffuse positive signal throughout the photoreceptor layer [40].

*ZWINT* directly specifies the localization of *ZW10* (Zeste White 10) to kinetochores and is essential for mitotic checkpoint signaling, as cells lacking *ZWINT* fail to arrest in mitosis when exposed to microtubule inhibitors, yielding interphase cells with multinuclei [41]. Musio et al. [42] found that inhibition of *INCENP*, *ZWINT*, and *ZW10* resulted in mitotic cells characterized by centromere separation, chromosome aneuploidy, and micronuclei formation, with chromosome morphology similar to that found in Roberts syndrome. The profound effects of *ZWINT* disruption made *ZWINT* a less compelling candidate gene and gene expression patterns of *ZWINT* in ocular tissues are unknown.

Direct sequencing of the coding regions of either gene did not reveal myopia-implicated mutations. Novel SNPs observed with both genes were submitted to the NCBI dbSNP database. Observed frequencies were submitted for known SNPs. Though there are no other known genes in this region, there is one predicted gene, *AL834134*, which encodes for the hypothetical protein DKFZp667A1711 of unknown function.

We also cannot rule out the possibility of genetic heterogeneity within this family. As mentioned earlier, it appears that the family is made of two distinct branches joined by marriage, so it is quite possible that the 1p36.32-1p36.31 locus (LOD=1.330) is involved in the high-grade myopia of the left branch of the family (affected haplotype with crosshatching) and the 10q21.1 locus is involved in the high-grade myopia of the right branch (affected haplotype in solid black). The small number of affected individuals in one branch may explain the lack of a significant LOD score for the 1p36.32-1p36.31 locus.

In summary, we have mapped a presumptive genetic locus for high-grade myopia. Mutational characterization of the remaining predicted gene in this region may provide additional insight into the molecular mechanisms underlying the development of high-grade myopia and into the regulation of eye growth. However, it is important to note that certain assumptions have been made in the determination of this locus, and further evidence of linkage of myopia to the 10q21.1 region should be sought out.
